# Drug resistance prediction for *Mycobacterium tuberculosis* with reference graphs

**DOI:** 10.1099/mgen.0.001081

**Published:** 2023-08-08

**Authors:** Michael B. Hall, Leandro Lima, Lachlan J. M. Coin, Zamin Iqbal

**Affiliations:** ^1^​ European Molecular Biology Laboratory, European Bioinformatics Institute, Hinxton, Cambridgeshire, UK; ^2^​ Department of Microbiology and Immunology, Peter Doherty Institute for Infection and Immunity, The University of Melbourne, Melbourne, Australia

**Keywords:** genome graphs, reference graphs, drug resistance prediction, *Mycobacterium tuberculosis*, benchmark, software

## Abstract

Tuberculosis is a global pandemic disease with a rising burden of antimicrobial resistance. As a result, the World Health Organization (WHO) has a goal of enabling universal access to drug susceptibility testing (DST). Given the slowness of and infrastructure requirements for phenotypic DST, whole-genome sequencing, followed by genotype-based prediction of DST, now provides a route to achieving this. Since a central component of genotypic DST is to detect the presence of any known resistance-causing mutations, a natural approach is to use a reference graph that allows encoding of known variation. We have developed DrPRG (Drug resistance Prediction with Reference Graphs) using the bacterial reference graph method Pandora. First, we outline the construction of a *Mycobacterium tuberculosis* drug resistance reference graph. The graph is built from a global dataset of isolates with varying drug susceptibility profiles, thus capturing common and rare resistance- and susceptible-associated haplotypes. We benchmark DrPRG against the existing graph-based tool Mykrobe and the haplotype-based approach of TBProfiler using 44 709 and 138 publicly available Illumina and Nanopore samples with associated phenotypes. We find that DrPRG has significantly improved sensitivity and specificity for some drugs compared to these tools, with no significant decreases. It uses significantly less computational memory than both tools, and provides significantly faster runtimes, except when runtime is compared to Mykrobe with Nanopore data. We discover and discuss novel insights into resistance-conferring variation for *M. tuberculosis* – including deletion of genes *katG* and *pncA* – and suggest mutations that may warrant reclassification as associated with resistance.

## Data Summary

The authors confirm all supporting data, code and protocols have been provided within the article or through supplementary data files.

The software method presented in this work, DrPRG, is freely available from GitHub under an MIT licence at https://github.com/mbhall88/drprg. We used commit 9492 f25 for all results via a Singularity [1] container from the URI docker://quay.io/mbhall88/drprg:9492 f25.

All code used to generate results for this study is available on GitHub at https://github.com/mbhall88/drprg-paper. All data used in this work are freely available from the SRA/ENA/DRA and a copy of the datasheet with all associated phenotype information can be downloaded from the archived repository at https://doi.org/10.5281/zenodo.7819984 or found in the previously mentioned GitHub repository.

The *Mycobacterium tuberculosis* index used in this work is available to download through DrPRG via the command drprg index --download mtb@20230308 or from GitHub at https://github.com/mbhall88/drprg-index.

**Impact Statement**

*
Mycobacterium tuberculosis* is the bacterium responsible for tuberculosis (TB). TB is one of the leading causes of death worldwide; before the coronavirus pandemic it was the leading cause of death from a single pathogen. Drug-resistant TB incidence has recently increased, making the detection of resistance even more vital. In this study, we develop a new software tool to predict drug resistance from whole-genome sequence data of the pathogen using new reference graph models to represent a reference genome. We evaluate it on *M. tuberculosis* against existing tools for resistance prediction and show improved performance. Using our method, we discover new resistance-associated variations and discuss reclassification of a selection of existing mutations. As such, this work contributes to TB drug resistance diagnostic efforts. In addition, the method could be applied to any bacterial species, so is of interest to anyone working on antimicrobial resistance.

## Introduction

Human industrialization of antibiotic production and use over the last 100 years has led to a global rise in the prevalence of antibiotic-resistant bacterial strains. The phenomenon was even observed within patients in the first clinical trial of streptomycin as a drug for tuberculosis (TB) in 1948 [2], and indeed as every new drug class has been introduced, so has resistance followed. Resistance mechanisms are varied, and can be caused by point mutations at key loci (e.g. binding sites of drugs [3, 4]), frameshifts rendering a gene non-functional [5], horizontal acquisition of new functionality via a new gene [6], or upregulation of efflux pumps to reduce the drug concentration within the cell [7].

Phenotypic and genotypic methods for detecting reduced susceptibility to drugs play complementary roles in clinical microbiology. Carefully defined phenotypic assays are used to give (semi)quantitative or binary measures of drug susceptibility; these have the benefit of being experimental, quantitative measurements, and can detect resistance caused by hitherto unknown mechanisms. Prediction of drug resistance from genomic data has different advantages. Detection of a single-nucleotide polymorphism (SNP) is arguably more consistent than a phenotypic assay, as it is not affected by whether the resistance it causes is near some threshold defining a resistant/susceptible boundary. Additionally, combining sequence datasets from different laboratories is more reliable than combining different phenotypic datasets, and using sequence data allows one to detect informative genetic changes (e.g. a tandem expansion of a single gene to form an array, thus increasing dosage). More subtly, defining the cut-off to separate resistant from susceptible is only simple when the minimum inhibitory concentration distribution is a simple bimodal distribution; in reality it is sometimes a convolution of multiple distributions caused by different mutations, and genetic data are sometimes needed to deconvolve the data and choose a threshold [8, 9].

The key requirement for a genomic predictor is to have an encodable understanding of the genotype-to-phenotype map. Research has focused on clinically important pathogens, primarily *Escherichia coli *, *Klebsiella pneumoniae *, *Salmonella enterica *, *Pseudomonas aeruginosa* and *Mycobacterium tuberculosis* (MTB). The challenges differ across species; almost all bacterial species are extremely diverse, with non-trivial pan-genomes and considerable horizontal gene transfer causing transmission of resistance genes [10]. In these cases, species are so diverse that detection of chromosomal SNPs is heavily affected by reference bias [11]. Furthermore, there is an appreciable proportion of resistance that is not currently explainable through known SNPs or genes [12–14]. At the other extreme, MTB has almost no accessory genome, and no recombination or plasmids [15]. Resistance appears to be caused entirely by mutations, insertion/deletions (indels) and rare structural variants, and simple sets of rules (‘if any of these mutations are present, or any of these genes inactivated, the sample is resistant’) work well for most drugs [16]. MTB has an exceptionally slow growth rate, meaning that culture-based drug susceptibility testing (DST) is slow (2–4 weeks, depending on media), and therefore sequencing is faster [17]. As part of the end TB strategy, the World Health Organization (WHO) strives towards universal access to DST [18], defining target product profiles for molecular diagnostics [19, 20] and publishing a catalogue of high-confidence resistance mutations intended to provide a basis for commercial diagnostics and future research [16]. There was a strong community-wide desire to integrate this catalogue into software for genotypic resistance prediction, although independent benchmarking confirmed that there was still need for improvement [12]. Hence, there is a continuing need to improve the understanding of the genetic basis of resistance and integrate it into software for genotypic DST.

In this paper we develop and evaluate a new software tool for genotypic DST for MTB, built on a generic framework that could be used for any bacteria. Several tools have been developed previously [21–25]. Of these, only Mykrobe and TBProfiler work on Illumina and Nanopore data, and both have been heavily evaluated previously [22, 23, 26, 27] – so we benchmark against these. Mykrobe uses de Bruijn graphs to encode known resistance alleles and thereby achieves high accuracy even on indel calls with Nanopore data [27]. However, it is unable to detect novel alleles in known resistance genes, nor to detect gene truncation or deletion, which would be desirable. TBProfiler is based on mapping and variant calling (by default using Freebayes [28]) and detects gene deletions using Delly [29].

Our new software, called DrPRG (Drug resistance Prediction with Reference Graphs), builds on newer pan-genome technology than Mykrobe [11] using an independent graph for each gene in the catalogue, which makes it easier to go back and forth between VCF and the graph. To build an index, it takes as input a catalogue of resistant variants (a simple four-column TSV file), a file specifying expert rules (e.g. any missense variant between codons X and Y in gene Z causes resistance to drug W) and a VCF of population variation in the genes of interest. This allows it to easily incorporate the current WHO-endorsed catalogue [16], which is conservative, and for the user to update the catalogue or rules with minimal effort. Finally, to provide resistance predictions, it takes a prebuilt index (an MTB one is currently provided) and sequencing reads (FASTQ).

We describe the DrPRG method, and to evaluate it, gather the largest MTB dataset of sequencing data with associated phenotype information and reveal novel insights into resistance-determining mutations for this species.

## Methods

DrPRG is a command-line software tool implemented in the Rust programming language. There are two main subcommands: *build* for building a reference graph and associated index files, and *predict* for producing genotypic resistance predictions from sequencing reads and an index (from *build*).

### Constructing a resistance-specific reference graph and index

The *build* subcommand of DrPRG requires a variant call format (VCF) file of variants from which to build a reference graph, a catalogue of mutations that confer resistance or susceptibility for one or more drugs and an annotation (GFF) and FASTA file of the reference genome.

For this work, we used the reference and annotation for the MTB strain H37Rv (accession NC_000962.3) and the default mutation catalogue from Mykrobe (v0.12.1) [12, 26].

To ensure the reference graph is not biased towards a particular lineage or susceptibility profile, we selected samples from a VCF of 15 211 global MTB samples [30]. We randomly chose 20 samples from each lineage 1 through 4, as well as 20 samples from all other lineages combined. In addition, we included 17 clinical samples representing MTB global diversity (lineages 1–6) [31, 32] to give a total of 117 samples. In the development phase of DrPRG we also found it necessary to add some common mutations not present in these 117 samples; as such, we added 48 mutations to the global VCF (these mutations were selected as they were the most common minor allele-causing mutations that were not in the reference graph and are listed in the archived repository – see Data Summary). We did not add all catalogue mutations as there is a saturation point for mutation addition to a reference graph, and beyond this point, performance begins to decay (see Sections S1 and S2, available in the online version of this article and [33]).

The *build* subcommand turns this VCF into a reference graph by extracting a consensus sequence for each gene and sample. We use just those genes that occur in the mutation catalogue and include 100 bases flanking the gene. A multiple sequence alignment is constructed for each gene from these consensus sequences with MAFFT (v7.505) [34, 35] and then a reference graph is constructed from these alignments with make_prg (v0.4.0) [11]. The final reference graph is then indexed with Pandora [11].

### Genotypic resistance prediction

Genotypic resistance prediction of a sample is performed by the *predict* subcommand of DrPRG. It takes an index produced by the *build* command (see Constructing a resistance-specific reference graph and index) and sequencing reads – Illumina or Nanopore are accepted. To generate predictions, DrPRG discovers novel variants (Pandora), adds these to the reference graph (make_prg and MAFFT) and then genotypes the sample with respect to this updated graph (Pandora). The genotyped VCF is filtered such that we ignore any variant with fewer than three reads supporting it and require a minimum of 1 % read depth on each strand (Section S2). Next, each variant is compared to the catalogue. If an alternative allele has been called that corresponds with a catalogue variant, resistance (‘R’) is noted for the drug(s) associated with that mutation. If a variant in the VCF matches a catalogue mutation, but the genotype is null (‘.’), we mark that mutation, and its associated drug(s), as failed (‘F’). Where an alternative allele call does not match a mutation in the catalogue, we produce an unknown (‘U’) prediction for the drug(s) that have a known resistance-conferring mutation in the relevant gene.

DrPRG also has the capacity to detect minor alleles and call minor resistance (‘r’) or minor unknown (‘u’) in such cases. Minor alleles are called when a variant (that has passed the above filtering) is genotyped as being the susceptible (reference) allele, but there is also read depth on the resistant (alternate) allele above a given minor allele frequency parameter (--maf; default is 0.1 for Illumina data). Minor allele calling is turned off by default for Nanopore data, as we found it led to a drastic increase in the number of false-positive calls (this is also the case for Mykrobe and TBProfiler).

When building the index for DrPRG and making predictions, we also accept a file of ‘expert rules’ for calling variants of a certain class. A rule is associated with a gene, an optional position range, a variant type and the drug(s) that rule confers resistance to. Currently supported variant types are missense, nonsense, frameshift and gene absence.

The output of running *predict* is a VCF file of all variants in the graph and a JSON file of resistance predictions for each drug in the index, along with the mutation(s) supporting that prediction and a unique identifier to find that variant in the VCF file (see Section S3 for an example). The reference graph gene presence/absence (as determined by Pandora) is also listed in the JSON file.

### Benchmark

We compare the performance of DrPRG against Mykrobe (v0.12.1) [26] and TBProfiler (v4.3.0) [22] for MTB drug resistance prediction. Mykrobe is effectively a predecessor of DrPRG; it uses genome graphs, in the form of de Bruijn graphs, to construct a graph of all mutations in a catalogue and then genotypes the reads against this graph. TBProfiler is a more traditional approach which aligns reads to a single reference genome and calls variants from that via aligned haplotype sequences.

A key part of such a benchmark is the catalogue of mutations, as this generally accounts for the majority of differences between tools [26]. As such, we use the same catalogue for all tools to ensure that any differences are method-related and not catalogue disparities. The catalogue we chose is the default one provided in Mykrobe [12]. It is a combination of the catalogue described in Hunt *et al.* [26] and the category 1 and 2 mutation and expert rules from the 2021 WHO catalogue [16]. This catalogue contains mutations for 14 drugs: isoniazid, rifampicin, ethambutol, pyrazinamide, levofloxacin, moxifloxacin, ofloxacin, amikacin, capreomycin, kanamycin, streptomycin, ethionamide, linezolid and delamanid.

We used Mykrobe and TBProfiler with default parameters, except for a parameter in each indicating the sequencing technology of the data as Illumina or Nanopore and the TBProfiler option to not trim data (as we do this in quality control).

We compare the prediction performance of each program using sensitivity and specificity. To calculate these metrics, we consider a true positive (TP) and true negative (TN) as a case where a program calls resistant and susceptible, respectively, and the phenotype agrees; a false positive (FP) as a resistant call by a program but a susceptible phenotype, with false negatives (FNs) being the inverse of FPs. We only present results for drugs in the catalogue and where at least 10 samples had phenotypic data available.

To benchmark the runtime and memory usage of each tool, we used the Snakemake benchmark feature within our analysis pipeline [36].

### Datasets

We gathered various MTB datasets where whole-genome sequencing data (Nanopore or Illumina) were available from public repositories (ENA/SRA/DRA) and associated phenotypes were accessible for at least one drug present in our catalogue [16, 27, 37–49].

All data were downloaded with fastq-dl (v1.1.1; https://github.com/rpetit3/fastq-dl).

### Quality control

All downloaded Nanopore fastq files had adapters trimmed with porechop (v0.2.4; https://github.com/rrwick/Porechop), with the option to discard any reads with an adapter in the middle, and any reads with an average quality score below 7 were removed with nanoq (v0.9.0) [50]. Illumina reads were preprocessed with fastp (v0.23.2) [51] to remove adapter sequences, trim low-quality bases from the ends of the reads, and remove duplicate reads and reads shorter than 30 bp.

Sequencing reads were decontaminated as described by Hall *et al.* [27] and Walker *et al.* [16]. Briefly, sequenced reads were mapped to a database of common sputum contaminants and the MTB reference genome (H37Rv; accession NC_000962.3) [52], only keeping those reads where the best mapping was to H37Rv.

After quality control, we removed any sample with an average read depth <15, or where more than 5 % of the reads mapped to contaminants.

Lineage information was extracted from the TBProfiler results (see Benchmark).

### Statistical analysis

We used a Wilcoxon rank-sum paired data test from the Python library SciPy [53] to test for significant differences in runtime and memory usage between the three prediction tools.

The sensitivity and specificity confidence intervals were calculated with a Wilson’s score interval with a coverage probability of 95 %.

## Results

**Fig. 1.**
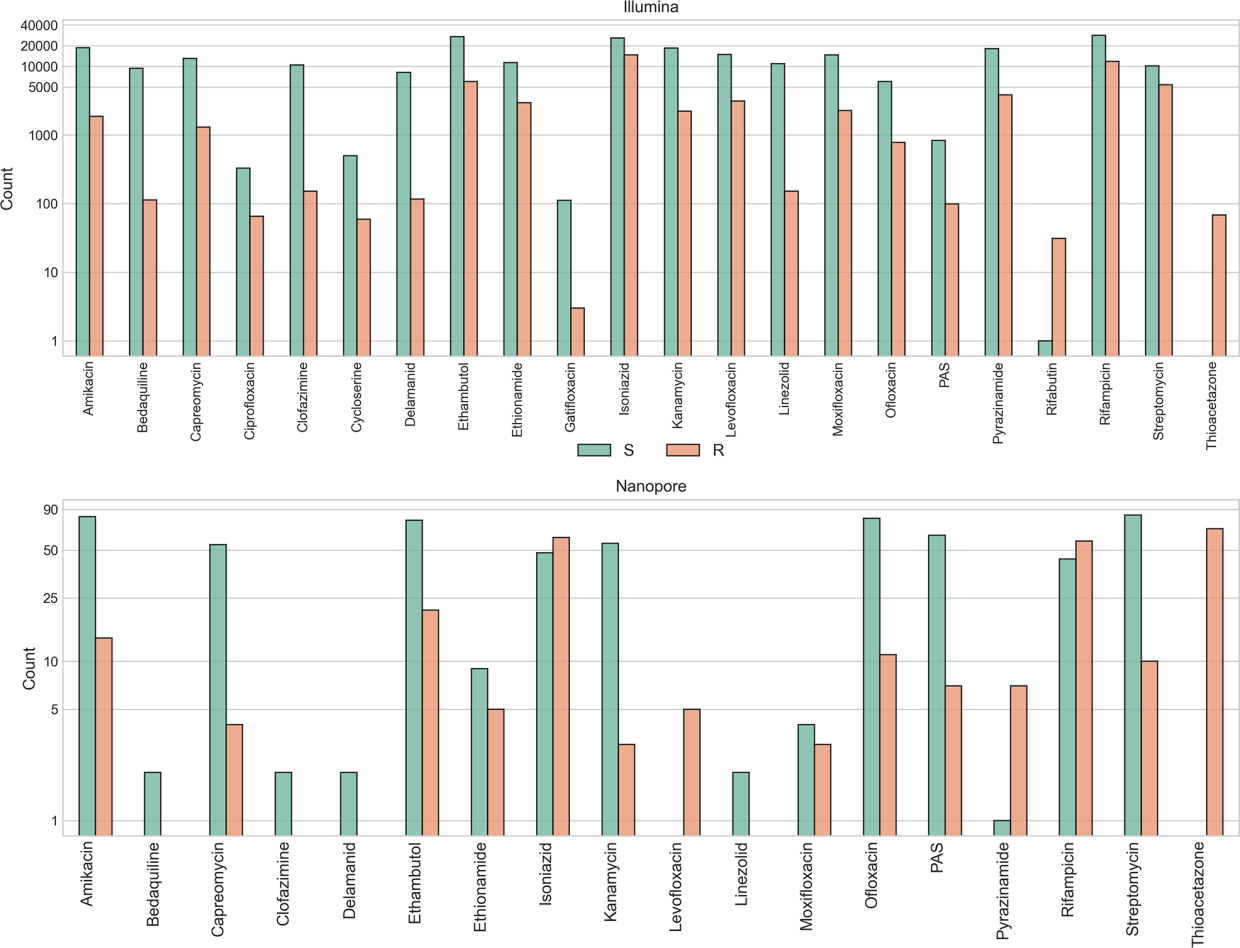
Drug phenotype counts for Illumina (upper) and Nanopore (lower) datasets. Bars are stratified and coloured by whether the phenotype is resistant (R; orange) or susceptible (S; green). Note, the *y*-axis is log-scaled. PAS para-aminosalicylic acid.

To benchmark DrPRG, Mykrobe and TBProfiler, we gathered an Illumina dataset of 45 702 MTB samples with a phenotype for at least 1 drug. After quality control (see Quality control), this number reduced to 44 709. In addition, we gathered 142 Nanopore samples, of which 138 passed quality control. In Fig. 1 we show all available drug phenotypes for those interested in the dataset, although our catalogue does not offer predictions for all drugs listed (see Benchmark). Lineage counts for all samples that passed quality control and have a single, major lineage call can be found in Table 1.

**Table 1.** Lineage counts from the Illumina and Nanopore datasets, covering the main lineages 1–9 (L1–L9) and the three livestock-associated lineages (La1–La3), as defined in [80]

Lineage	Illumina	Nanopore
La1	239	0
La2	7	0
La3	71	0
L1	3907	32
L2	12 870	38
L3	5803	9
L4	20 731	59
L5	63	0
L6	78	0
L7	3	0
L9	1	0

### Sensitivity and specificity performance

**Fig. 2.**
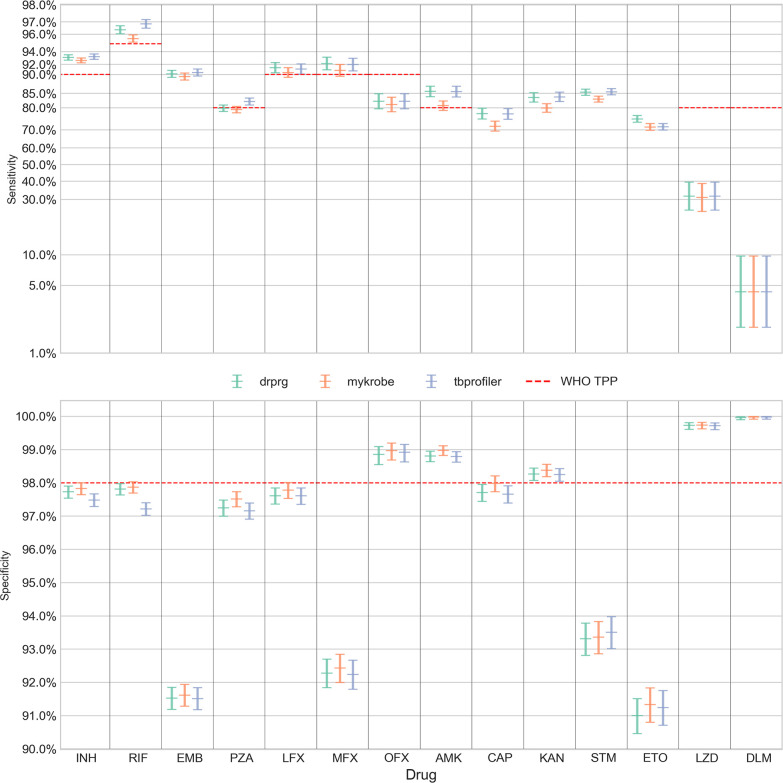
Sensitivity (upper panel; *y*-axis) and specificity (lower panel; *y*-axis) of resistance predictions for different drugs (*x*-axis) from Illumina data. Error bars are coloured by prediction tool. The central horizontal line in each error bar is the sensitivity/specificity and the error bars represent the 95 % confidence interval. Note, the sensitivity panel’s *y*-axis is logit-scaled. This scale is similar to a log scale close to zero and to one (100%), and almost linear around 0.5 (50 %). The red dashed line in each panel represents the minimal standard WHO target product profile (TPP; where available) for next-generation drug susceptibility testing for sensitivity and specificity. INH isoniazid; RIF, rifampicin; EMB, ethambutol; PZA, pyrazinamide; LFX, levofloxacin; MFX, moxifloxacin; OFX, ofloxacin; AMK, amikacin; CAP, capreomycin; KAN, kanamycin; STM, streptomycin; ETO, ethionamide; LZD, linezolid; DLM, delamanid.

**Fig. 3.**
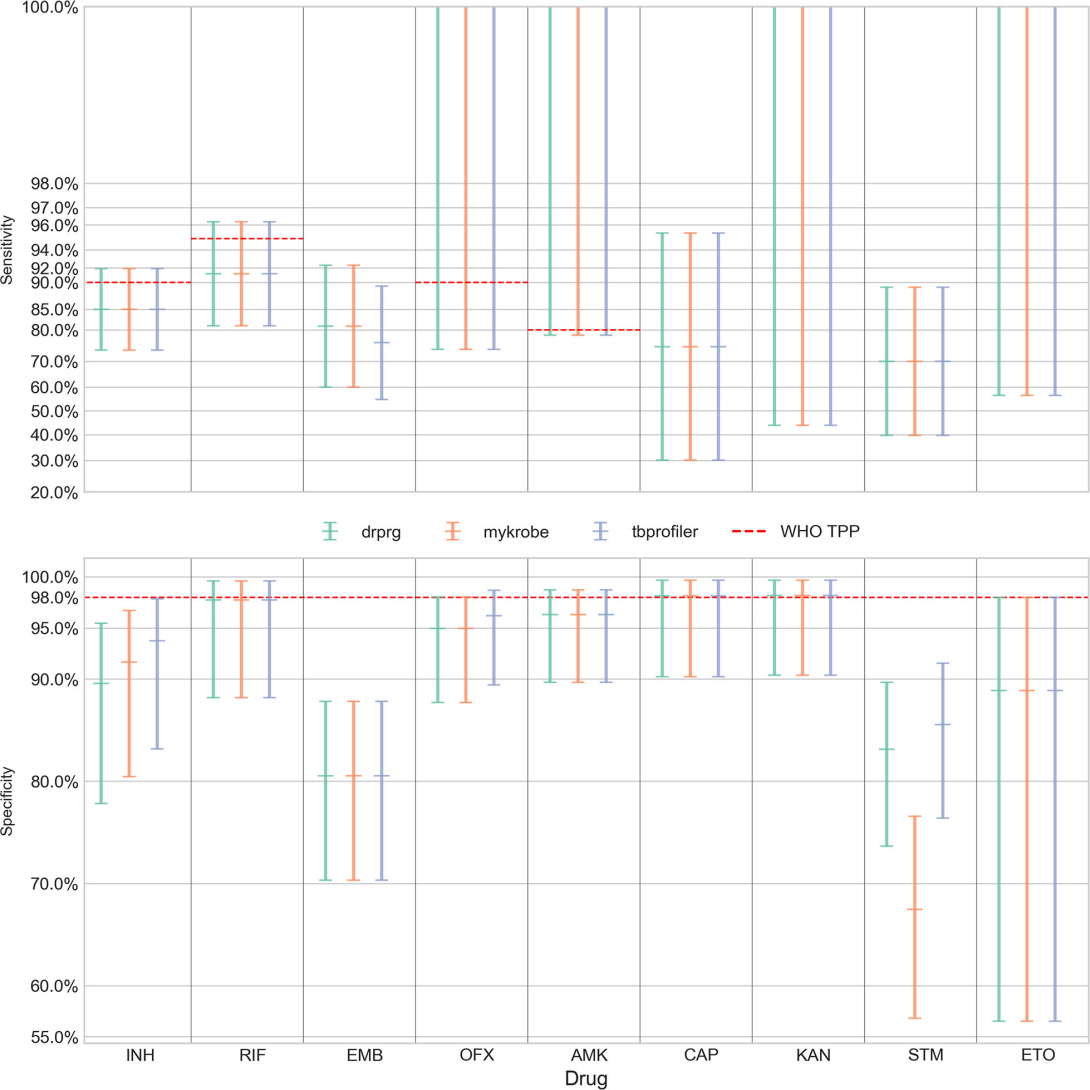
Sensitivity (upper panel; *y*-axis) and specificity (lower panel; *y*-axis) of resistance predictions for different drugs (*x*-axis) from Nanopore data. Error bars are coloured by prediction tool. The central horizontal line in each error bar is the sensitivity/specificity and the error bars represent the 95 % confidence interval. Note, the sensitivity panel’s *y*-axis is logit-scaled. This scale is similar to a log scale close to zero and to one (100%), and almost linear around 0.5 (50 %). The red dashed line in each panel represents the minimal standard WHO target product profile (TPP; where available) for next-generation drug susceptibility testing for sensitivity and specificity. INH isoniazid; RIF, rifampicin; EMB, ethambutol; OFX, ofloxacin; AMK, amikacin; CAP, capreomycin; KAN, kanamycin; STM, streptomycin; ETO, ethionamide.

We present the sensitivity and specificity results for Illumina data in Fig. 2 and Table S2 and the Nanopore data in Fig. 3 and Table S3.

When comparing DrPRG’s performance to that of Mykrobe and TBProfiler, we look for instances where the confidence intervals do not overlap, indicating a significant difference. With Illumina data (Fig. 2 and Table S), DrPRG achieves significantly greater sensitivity than Mykrobe for rifampicin [96.4 % (96.0–96.7) vs 95.6 % (95.2–95.9)], streptomycin [85.3 % (84.4–86.3) vs 83.1 % (82.1–84.1)], amikacin [85.6 % (83.9–87.1) vs 80.8 % (78.9–82.5)], capreomycin [77.5 % (75.2–79.7) vs 71.8 % (69.3–74.1)], kanamycin [83.7 % (82.1–85.2) vs 79.9 % (78.2–81.5)] and ethionamide [75.2 % (73.7–76.8) vs 71.4 % (69.7–73.0)], with no significant difference for all other drugs. In terms of sensitivity, there was no significant difference between DrPRG and TBProfiler except for ethionamide, where DrPRG was significantly more sensitive [75.2 % (73.7–76.8) vs 71.5 % (69.8–73.1)]. For specificity, there was no significant difference between the tools except that DrPRG and Mykrobe were significantly better than TBProfiler for rifampicin [97.8 % (97.6–98.0) vs 97.2 % (97.0–97.4)]. There was no significant difference in sensitivity or specificity for any drug with Nanopore data.

In both figures, we show the minimal requirements from the WHO target product profiles for the sensitivity and specificity of genotypic drug susceptibility testing [19] as red dashed lines. Note, a sensitivity target is not specified by the WHO for ethambutol (EMB), capreomycin (CAP), kanamycin (KAN), streptomycin (STM), or ethionamide (ETO). For Illumina data, all tools’ predictions for rifampicin, isoniazid, levofloxacin, moxifloxacin and amikacin are above the sensitivity minimal requirement target. TBProfiler also exceeds the target for pyrazinamide, which DrPRG misses by 0.2 %. No drug’s sensitivity target was achieved with Nanopore data. For specificity, the tools are all very similar and either exceed or fall below the threshold together (see Fig. 2). The target of >98 % is only met by all tools with Illumina data for ofloxacin, amikacin, linezolid and delamanid. Mykrobe also exceeds the target for capreomycin. As such, amikacin is the only drug where both sensitivity and specificity performance exceed the minimal requirement of the WHO target product profiles. Only capreomycin and kanamycin specificity targets are exceeded (by all tools) with Nanopore data.

However, for Illumina data, we did find that likely sample swaps or phenotype instability [54] could lead to some drugs being on the threshold of the WHO target product profiles. If we excluded samples where all three tools make a FP call for the strong isoniazid and rifampicin resistance-conferring mutations *katG* S315T (*n*=152) and *rpoB* S450L (*n*=119) [16], respectively, all three tools would exceed the isoniazid specificity target of 98 % – thus meeting both sensitivity and specificity targets for isoniazid. In addition, DrPRG and Mykrobe would meet the rifampicin specificity target of 98 % – leading to both targets being met for rifampicin for these two tools. As previously reported [54, 55], we also found considerable instability in the ethambutol result caused by *embB* mutations M306I (*n*=827) and M306V (*n*=519) being called for phenotypically susceptible samples (FP) by all three tools. Other frequent consensus FP calls included *fabG1* c-15t, which is associated with ethionamide (*n*=441) and isoniazid (*n*=241) resistance, and *rrs* a1401g, which is associated with resistance to capreomycin (*n*=241), amikacin (*n*=70) and kanamycin (*n*=48). In addition, there were common false positives from *gyrA* mutations A90V and D94G, which are associated with resistance to the fluoroquinolones levofloxacin (*n*=108 and *n*=70, respectively), moxifloxacin (*n*=419 and *n*=349) and ofloxacin (*n*=19 and *n*=17), and are known to cause heteroresistance and minimum inhibitory concentrations (MICs) close to the critical concentration threshold [56–58].

### Evaluation of potential additions to the WHO catalogue

False negatives are much harder to investigate because it is not known which mutation(s) were missed, as they are presumably not in the catalogue if all tools failed to make a call. However, looking through those FNs where DrPRG makes an ‘unknown’ resistance call, we note some potential mutations that may require reclassification or inclusion in the WHO catalogue. For delamanid FNs, we found five different nonsense mutations in the *ddn* gene in seven samples – W20* (*n*=2), W27* (*n*=1), Q58* (*n*=1), W88* (*n*=2) and W139* (*n*=1) – none of which occurred in susceptible samples. We also found 13 pyrazinamide FN cases with a nonstop (stop–loss) mutation in *pncA*; this mutation type was also seen in 2 susceptible samples. Another *pncA* mutation, T100P, was also observed in 10 pyrazinamide FN samples and no susceptible samples. T100P only appears once in the WHO catalogue data (‘solo’ in a resistant sample). As such, it was given a grading of uncertain significance. As our dataset includes those samples in the WHO catalogue dataset, this means an additional nine isolates have been found with this mutation, indicating that this may warrant an upgrade to ‘associated with resistance’.

We found an interesting case of allele combinations, where nine ethambutol FN samples have the same two *embA* mutations, c-12a and c-11a, and *embB* mutation P397T; this combination is only seen in two susceptible samples. Interestingly, *embB* P397T and *embA* c-12a do not appear in the WHO catalogue, but have been described as causing resistance previously [59]. Three *katG* mutations were also detected in isoniazid FN cases. First, G279D occurs in eight missed resistance samples and no susceptible cases. This mutation is graded as ‘uncertain significance’ in the WHO catalogue and was seen solo in four resistant samples in that data. Singh *et al.* performed a protein structural analysis caused by this mutation and found that it produced ‘an undesirable effect on the functionality of the protein’ [60]. Second, G699E occurs in eight FN samples and no susceptible cases, but has a WHO grading of ‘uncertain significance’ based on six resistant isolates; thus, we add two extra samples to that count. And third, N138H occurs in 14 FN samples and 1 susceptible sample. In seven of these cases, it co-occurs with *ahpC* mutations t-75g (*n*=2) and t-76a (*n*=5). This mutation only occurs in 3 resistant isolates in the WHO catalogue dataset, giving it an uncertain significance, but we add a further 11 cases. This mutation has been found to cause a high isoniazid MIC and to be associated with resistance [61, 62].

### Detection of large deletions

**Fig. 4.**
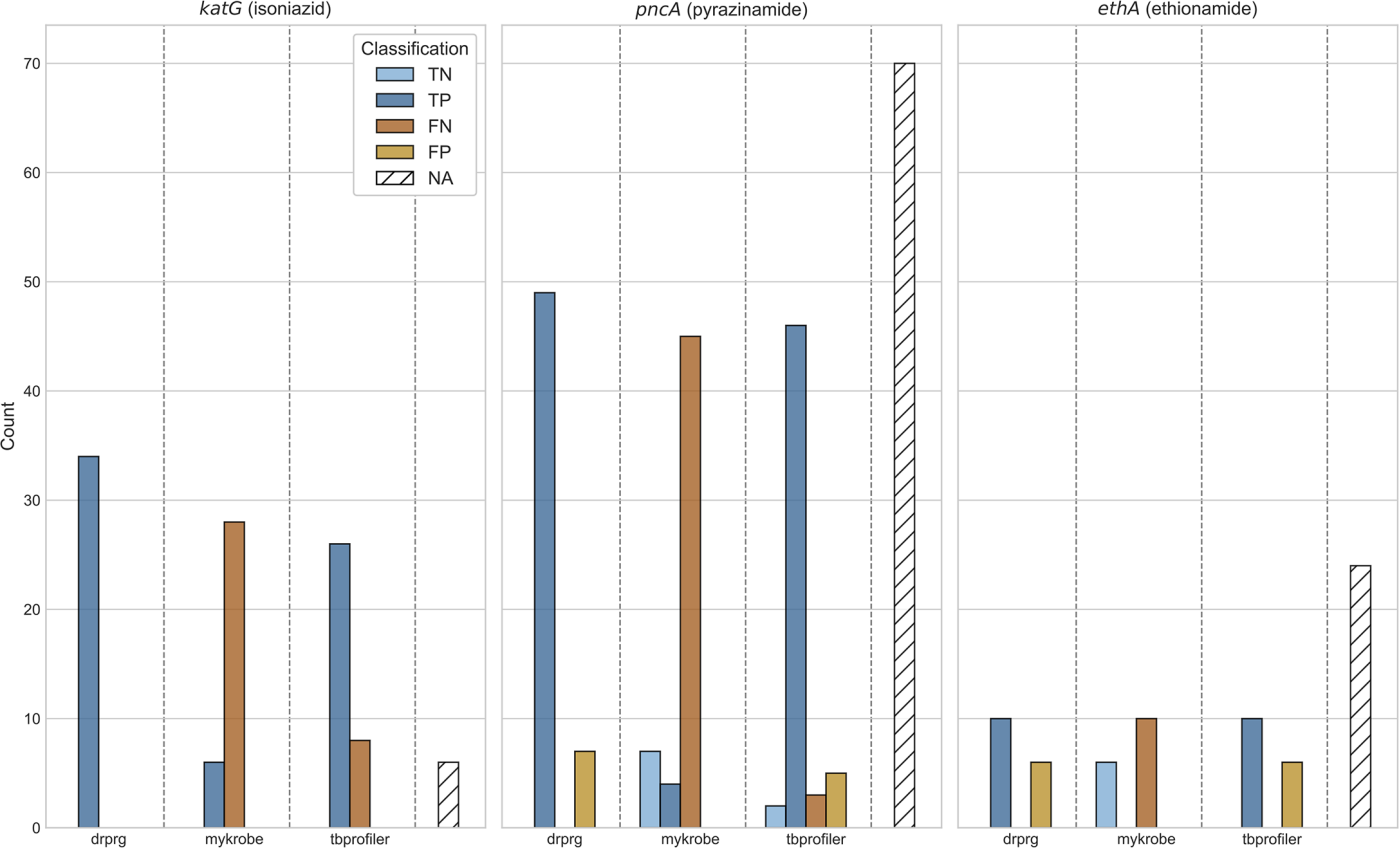
Impact of gene deletion on resistance classification. The title of each subplot indicates the gene and drug it effects. Bars are coloured by their classification and stratified by tool. Count (*y*-axis) indicates the number of gene deletions for that category. The NA bar (white with diagonal lines) indicates the number of samples with that gene deleted but no phenotype information for the respective drug. TP true positive; FN, false negative; TN, true negative; FP, false positive; NA, no phenotype available.

There are expert rules in the WHO catalogue that treat gene loss of function (any frameshift or nonsense mutation) in *katG*, *ethA*, *gid* and *pncA* as causing resistance for isoniazid, ethionamide, streptomycin and pyrazinamide, respectively [16]. Although examples of resistance caused by gene deletion are rare [63–67], with a dataset of this size (*n*=44 709), we can both evaluate these rules and compare the detection power of DrPRG and TBProfiler for identifying gene deletions (Mykrobe does not, although in principle it could). In total we found 206 samples where DrPRG and/or TBProfiler identified deletions of *ethA*, *katG*, or *pncA*. Although many of these isolates did not have phenotype information for the associated drug (*n*=100), the results are nevertheless striking (Fig. 4). Given the low false-positive rate of Pandora for gene absence detection [11], these no-phenotype samples provide insight into how often gene deletions are occurring in clinical samples.

Of the 34 isolates where *katG* was identified as being absent, and an isoniazid phenotype was available, all 34 were phenotypically resistant. DrPRG detected all 34 (100 % sensitivity) and TBProfiler identified 26 (76.5 % sensitivity). Deletions of *pncA* were detected in 56 isolates, of which 49 were phenotypically resistant. DrPRG detected 47 (95.9 % sensitivity) and TBProfiler detected 46 (93.9 % sensitivity). Lastly, *ethA* was found to be missing in 16 samples with an ethionamide phenotype, of which 10 were phenotypically resistant. Both DrPRG and TBProfiler correctly predicted all 10 (100 % sensitivity). No *gid* deletions were discovered. We note that the TP calls made by Mykrobe were due to it detecting large deletions that are present in the catalogue, which is understandable given that the whole gene is deleted.

We conclude that DrPRG is slightly more sensitive at detecting large deletions than TBProfiler (and Mykrobe) for *katG*, and equivalent for *pncA* and *ethA.* However, we note that the WHO expert rule, which predicts resistance to isolates missing specific genes, appears to be more accurate for *katG* (100 % of isolates missing the gene are resistant) than for *pncA* (87 % resistant) and *ethA* (62.5 % resistant).

### Runtime and memory usage benchmark

**Fig. 5.**
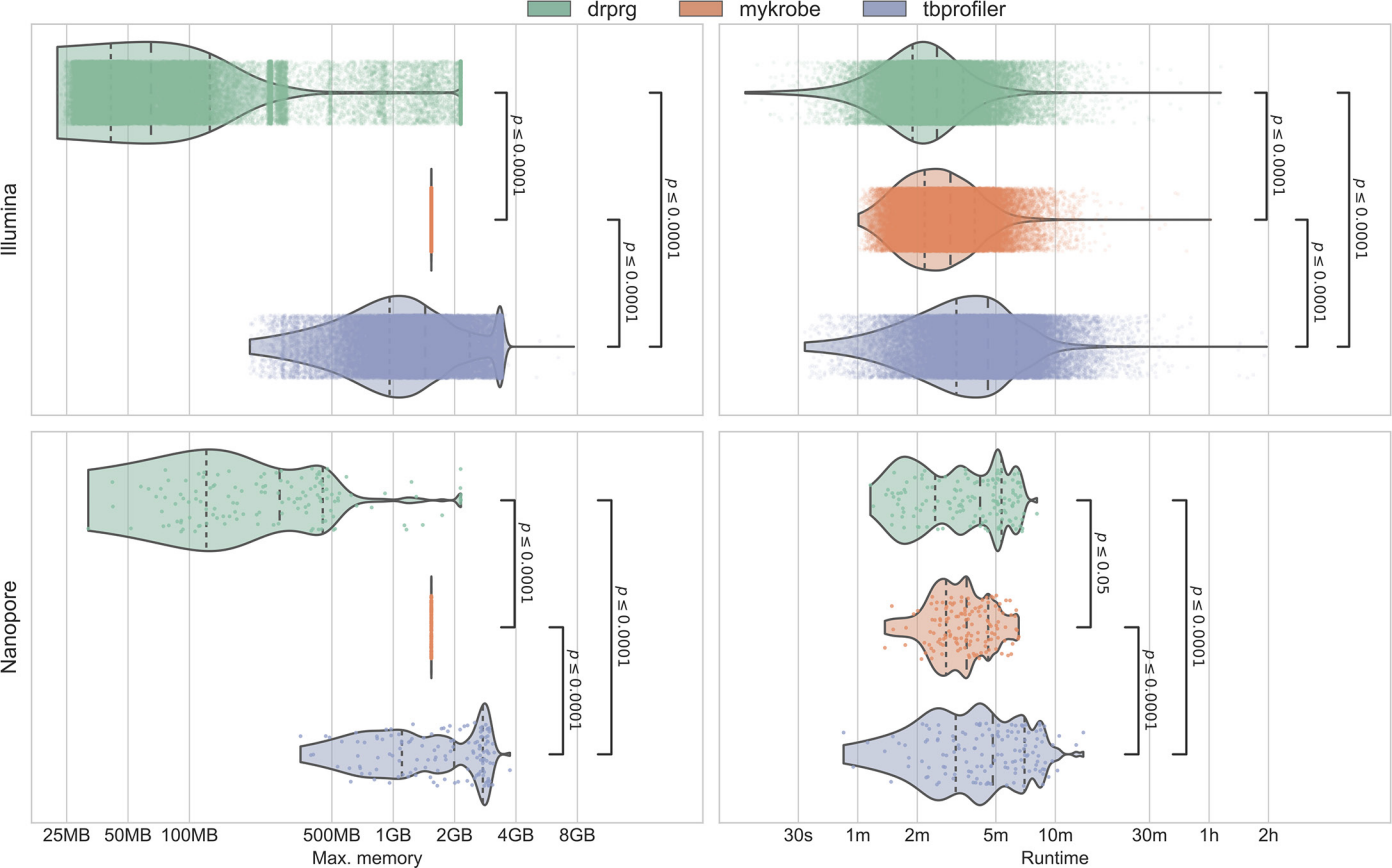
Benchmark of the maximum memory usage (left panels) and runtime (right panels) for Illumina (upper row) and Nanopore (lower row) data. Each point and violin is coloured by the tool, with each point representing a single sample. Statistical annotations are the result of a Wilcoxon rank-sum paired data test on each pair of tools. Dashed lines inside the violins represent the quartiles of the distribution. Note, the *x*-axis is log-scaled.

The runtime and peak memory usage of each program was recorded for each sample and are presented in Fig. 5. DrPRG (median 161 s) was significantly faster than both TBProfiler (307 s; *P*≤0.0001) and Mykrobe (230 s; *P*≤0.0001) with Illumina data. For Nanopore data, DrPRG (250 s) was significantly faster than TBProfiler (290 s; *P*≤0.0001), but significantly slower than Mykrobe (213 s; *P*=0.0347). In terms of peak memory usage, DrPRG (Illumina median peak memory 58 MB; Nanopore 277 MB) used significantly less memory than Mykrobe (1538 MB; 1538 MB) and TBProfiler (1463 MB; 1990 MB) for both Illumina and Nanopore data (*P*≤0.0001 for all comparisons).

## Discussion

The dominant paradigm for analysing genetic variation relies on a central idea: all genomes in a species can be described as minor differences from a single reference genome. However, this approach can be problematic or inadequate for bacteria, where there can be significant sequence divergence within a species. Reference graphs are an emerging solution to the reference bias issues implicit in the ‘single-reference’ model [11, 68, 69]. Such a graph represents variation at multiple scales within a population – e.g. nucleotide and locus level. The graph structure used in Pandora is gene (or locus) oriented, and allows natural support or analysing SNP and indel mutations and the presence/absence of genes.

In this work, we have presented a novel method for making drug resistance predictions with reference graphs. The method, DrPRG, requires only a reference genome and annotation, a catalogue of resistance-conferring mutations, a VCF of population variation from which to build a reference graph and (optionally) a set of rules for types of variants in specific genes that cause resistance. We apply DrPRG to the pathogen *M. tuberculosis *, for which there is a great deal of information on the genotype/phenotype relationship, and a great need to provide good tools that implement and augment current and forthcoming versions of the WHO catalogue. We illustrate the performance of DrPRG against two existing methods for drug resistance prediction – Mykrobe and TBProfiler.

We benchmarked the methods on a high-quality Illumina sequencing dataset with associated phenotype profiles for 44 709 MTB genomes; the largest known dataset to date [16]. All tools used the same catalogue and rules, and for most drugs there was no significant difference between the tools. However, DrPRG did have a significantly higher specificity than TBProfiler for rifampicin predictions, and sensitivity for ethionamide predictions. DrPRG’s sensitivity was also significantly greater than Mykrobe’s for rifampicin, streptomycin, amikacin, capreomycin, kanamycin and ethionamide. Evaluating detection of gene loss, we found that DrPRG was more sensitive to *katG* deletions than TBProfiler.

We also benchmarked using 138 Nanopore-sequenced MTB samples with phenotype information, but found no significant difference between the tools. This Nanopore dataset was quite small and therefore the confidence intervals were large for all drugs.

DrPRG also used significantly less memory than Mykrobe and TBProfiler on both Nanopore and Illumina data. In addition, the runtime of DrPRG was significant faster than both tools for Illumina data and faster than TBProfiler for Nanopore data. While the absolute values for memory and runtime for all tools mean that they could all easily run on common computers found in the types of institutions likely to run them, the differences for the Nanopore data warrant noting. As Nanopore data can be generated ‘in the field’, computational resource usage is critical. For example, in a recent collaboration of ours with the National Tuberculosis programme in Madagascar [27], Nanopore sequencing and analysis are regularly performed on a laptop, meaning that memory usage is sometimes a limiting factor. DrPRG’s median peak memory was 277 MB, meaning that it can comfortably be run on any laptop or other mobile computing device [70].

It is clear from the Illumina results that more work is needed to understand resistance-conferring mutations for delamanid and linezolid. However, we did find that nonsense mutations in the *ddn* gene appear likely to be resistance-conferring for delamanid – as has been noted previously [39, 71–73]. We also found a novel (likely) mechanism of resistance to pyrazinamide – a nonstop mutation in *pncA*. Phenotype instability in *embB* at codon 306 was also found to be the main driver in poor ethambutol specificity, as has been noted elsewhere [54, 55], indicating the need to further investigate cofactors that may influence the phenotype when mutations at this codon are present.

Gene absence/deletion detection allowed us to confirm that the absence of *katG* – a mechanism that is rare in clinical samples [63–66, 74] – is highly likely to confer resistance to isoniazid. Additionally, we found that the absence of *pncA* is likely to cause resistance to pyrazinamide, as has been noted previously [67]. One finding that requires further investigation is the variability in ethionamide phenotype when *ethA* is absent. We found that only 63 % of the samples with *ethA* missing and an ethionamide phenotype were resistant. Ang *et al.* have suggested that *ethA* deletion alone does not always cause resistance and there might be an alternative pathway via *mshA* [75].

Given the size of the Illumina dataset used in this work, the results provide a good marker of Illumina whole-genome sequencing’s ability to replace traditional phenotyping methods. With the catalogue used in this study, DrPRG meets the WHO’s target product profile for next-generation drug-susceptibility testing for both sensitivity and specificity for amikacin, and sensitivity only for rifampicin, isoniazid, levofloxacin and moxifloxacin. However, if we exclude cases where all tools call *rpoB* S450L or *katG* S315T for phenotypically susceptible samples (these are strong markers of resistance [16] and therefore we suspect sample swaps or phenotype error [76]), DrPRG also meets the specificity target product profile for rifampicin and isoniazid. For the other first-line drugs, ethambutol and pyrazinamide, ethambutol does not have a WHO target and DrPRG’s pyrazinamide sensitivity is 0.2 % below the WHO target (although the confidence interval spans the target), while the pyrazinamide specificity target is missed by 0.8 %.

The primary limitation of the DrPRG method relates to minor allele calls. DrPRG uses Pandora for novel variant discovery, combining a graph of known population variants (which can be detected at low frequency) with *de novo* detection of other variants if present at above ~50 % frequency. Thus, it can miss minor allele calls if the allele is absent from its reference graph. While this issue did not impact on most drugs, it did account for the majority of cases where DrPRG missed pyrazinamide-resistant calls (in *pncA*), but the other tools correctly called resistance. Unlike most other genes, where there are a relatively small number of resistance-conferring mutations, or they are localized to a specific region (e.g. the rifampicin resistance-determining region in *rpoB*), resistance-conferring mutations are numerous – with most being rare – and distributed throughout *pncA* [16, 77, 78]. Adding all of these mutations leads to decreased performance of the reference graph (Section S3 and [33]), and so improving minor allele calling for pyrazinamide remains a challenge we need to revisit in the future.

One final limitation is the small number of Nanopore-sequenced MTB isolates with phenotypic information. Increased Nanopore sequencing over time will provide better resolution of the overall sensitivity and specificity values and improve the methodological nuances of calling variants from this emerging, and continually changing, sequencing technology.

In conclusion, DrPRG is a fast, memory-frugal software program for predicting drug resistance. We showed that on MTB it performs as well as, or better than, two other commonly used tools for resistance prediction. We also collected and curated the largest dataset of MTB Illumina-sequenced genomes with phenotype information and hope this will benefit future work to improved genotypic drug susceptibility testing for this species. While we applied DrPRG to MTB in this study, it is a framework that is agnostic to species. MTB is likely one of the bacterial species with the least to gain from reference graphs, given its relatively conserved (closed) pan-genome compared to other common species [79]. As such, we expect the benefits and performance of DrPRG to improve as the openness of the species’ pan-genome increases [11], especially given its good performance on a reasonably closed pan-genome.

## Supplementary Data

Supplementary material 1

## References

[1] Kurtzer GM, Sochat V, Bauer MW (2017). Singularity: scientific containers for mobility of compute. PLoS One.

[2] Medical Research Council (1948). Streptomycin treatment of pulmonary tuberculosis: a medical research council Investigation. BMJ.

[3] Wengenack NL, Todorovic S, Yu L, Rusnak F (1998). Evidence for differential binding of isoniazid by *Mycobacterium tuberculosis* KatG and the isoniazid-resistant mutant KatG(S315T). Biochemistry.

[4] Hackbarth CJ, Kocagoz T, Kocagoz S, Chambers HF (1995). Point mutations in *Staphylococcus aureus* PBP 2 gene affect penicillin-binding kinetics and are associated with resistance. Antimicrob Agents Chemother.

[5] Esposito EP, Cervoni M, Bernardo M, Crivaro V, Cuccurullo S (2018). Molecular epidemiology and virulence profiles of colistin-resistant *Klebsiella pneumoniae* blood isolates from the hospital agency “Ospedale dei Colli,” Naples, Italy. Front Microbiol.

[6] Liu Y-Y, Wang Y, Walsh TR, Yi L-X, Zhang R (2016). Emergence of plasmid-mediated colistin resistance mechanism MCR-1 in animals and human beings in China: a microbiological and molecular biological study. Lancet Infect Dis.

[7] Maira-Litrán T, Allison DG, Gilbert P (2000). An evaluation of the potential of the multiple antibiotic resistance operon (mar) and the multidrug efflux pump acrAB to moderate resistance towards ciprofloxacin in *Escherichia coli* biofilms. J Antimicrob Chemother.

[8] Werngren J, Sturegård E, Juréen P, Ängeby K, Hoffner S (2012). Reevaluation of the critical concentration for drug susceptibility testing of *Mycobacterium tuberculosis* against pyrazinamide using wild-type MIC distributions and pncA gene sequencing. Antimicrob Agents Chemother.

[9] Schön T, Miotto P, Köser CU, Viveiros M, Böttger E (2017). *Mycobacterium tuberculosis* drug-resistance testing: challenges, recent developments and perspectives. Clin Microbiol Infect.

[10] McInerney JO, McNally A, O’Connell MJ (2017). Why prokaryotes have pangenomes. Nat Microbiol.

[11] Colquhoun RM, Hall MB, Lima L, Roberts LW, Malone KM (2021). Pandora: nucleotide-resolution bacterial pan-genomics with reference graphs. Genome Biol.

[12] Hall MB, Coin LJM (2022). Assessment of the 2021 WHO *Mycobacterium tuberculosis* drug resistance mutation catalogue on an independent dataset. Lancet Microbe.

[13] Mahfouz N, Ferreira I, Beisken S, von Haeseler A, Posch AE (2020). Large-scale assessment of antimicrobial resistance marker databases for genetic phenotype prediction: a systematic review. J Antimicrob Chemother.

[14] Hendriksen RS, Bortolaia V, Tate H, Tyson GH, Aarestrup FM (2019). Using genomics to track global antimicrobial resistance. Front Public Health.

[15] Godfroid M, Dagan T, Kupczok A (2018). Recombination signal in *Mycobacterium tuberculosis* stems from reference-guided assemblies and alignment artefacts. Genome Biol Evol.

[16] Walker TM, Miotto P, Köser CU, Fowler PW, Knaggs J (2022). The 2021 WHO catalogue of *Mycobacterium tuberculosis* complex mutations associated with drug resistance: a genotypic analysis. Lancet Microbe.

[17] Votintseva AA, Bradley P, Pankhurst L, Del Ojo Elias C, Loose M (2017). Same-day diagnostic and surveillance data for tuberculosis via whole-genome sequencing of direct respiratory samples. J Clin Microbiol.

[18] The end TB strategy (2015). Report No.: WHO/HTM/TB/2015.19. Available. https://www.who.int/publications/i/item/WHO-HTM-TB-2015.19.

[19] (2021). Target product profile for next-generation drug-susceptibility testing at peripheral centres. https://www.who.int/publications/i/item/9789240032361.

[20] MacLean EL-H, Miotto P, González Angulo L, Chiacchiaretta M, Walker TM (2023). Updating the WHO target product profile for next-generation *Mycobacterium tuberculosis* drug susceptibility testing at peripheral centres. PLoS Glob Public Health.

[21] Steiner A, Stucki D, Coscolla M, Borrell S, Gagneux S (2014). KvarQ: targeted and direct variant calling from fastq reads of bacterial genomes. BMC Genomics.

[22] Phelan JE, O’Sullivan DM, Machado D, Ramos J, Oppong YEA (2019). Integrating informatics tools and portable sequencing technology for rapid detection of resistance to anti-tuberculous drugs. Genome Med.

[23] Bradley P, Gordon NC, Walker TM, Dunn L, Heys S (2015). Rapid antibiotic-resistance predictions from genome sequence data for *Staphylococcus aureus* and *Mycobacterium tuberculosis*. Nat Commun.

[24] Kohl TA, Utpatel C, Schleusener V, De Filippo MR, Beckert P (2018). MTBseq: a comprehensive pipeline for whole genome sequence analysis of *Mycobacterium tuberculosis* complex isolates. PeerJ.

[25] Feuerriegel S, Schleusener V, Beckert P, Kohl TA, Miotto P (2015). PhyResSE: a web tool delineating *Mycobacterium tuberculosis* antibiotic resistance and lineage from whole-genome sequencing data. J Clin Microbiol.

[26] Hunt M, Bradley P, Lapierre SG, Heys S, Thomsit M (2019). Antibiotic resistance prediction for *Mycobacterium tuberculosis* from genome sequence data with Mykrobe. Wellcome Open Res.

[27] Hall MB, Rabodoarivelo MS, Koch A, Dippenaar A, George S (2023). Evaluation of Nanopore sequencing for *Mycobacterium tuberculosis* drug susceptibility testing and outbreak investigation: a genomic analysis. Lancet Microbe.

[28] Garrison E, Marth G (2012). Haplotype-based variant detection from short-read sequencing. arXiv.

[29] Rausch T, Zichner T, Schlattl A, Stütz AM, Benes V (2012). DELLY: structural variant discovery by integrated paired-end and split-read analysis. Bioinformatics.

[30] Ladner J, The CRyPTIC Consortium (2022). A data compendium associating the genomes of 12,289 *Mycobacterium tuberculosis* isolates with quantitative resistance phenotypes to 13 antibiotics. PLoS Biol.

[31] Chiner-Oms Á, Berney M, Boinett C, González-Candelas F, Young DB (2019). Genome-wide mutational biases fuel transcriptional diversity in the *Mycobacterium tuberculosis* complex. Nat Commun.

[32] Letcher B, Hunt M, Iqbal Z (2021). Gramtools enables multiscale variation analysis with genome graphs. Genome Biol.

[33] Pritt J, Chen N-C, Langmead B (2018). FORGe: prioritizing variants for graph genomes. Genome Biol.

[34] Katoh K, Frith MC (2012). Adding unaligned sequences into an existing alignment using MAFFT and LAST. Bioinformatics.

[35] Katoh K, Misawa K, Kuma K, Miyata T (2002). MAFFT: a novel method for rapid multiple sequence alignment based on fast Fourier transform. Nucleic Acids Res.

[36] Mölder F, Jablonski KP, Letcher B, Hall MB, Tomkins-Tinch CH (2021). Sustainable data analysis with Snakemake. F1000Res.

[37] Gröschel MI, Owens M, Freschi L, Vargas R, Marin MG (2021). GenTB: a user-friendly genome-based predictor for tuberculosis resistance powered by machine learning. Genome Med.

[38] Trisakul K, Nonghanphithak D, Chaiyachat P, Kaewprasert O, Sakmongkoljit K (2022). High clustering rate and genotypic drug-susceptibility screening for the newly recommended anti-tuberculosis drugs among global extensively drug-resistant *Mycobacterium tuberculosis* isolates. Emerg Microbes Infect.

[39] Battaglia S, Spitaleri A, Cabibbe AM, Meehan CJ, Utpatel C (2020). Characterization of genomic variants associated with resistance to bedaquiline and delamanid in naive *Mycobacterium tuberculosis* clinical strains. J Clin Microbiol.

[40] Huang H, Ding N, Yang T, Li C, Jia X (2019). Cross-sectional whole-genome sequencing and epidemiological study of multidrug-resistant *Mycobacterium tuberculosis* in China. Clin Infect Dis.

[41] Bainomugisa A, Lavu E, Hiashiri S, Majumdar S, Honjepari A (2018). Multi-clonal evolution of multi-drug-resistant/extensively drug-resistant *Mycobacterium tuberculosis* in a high-prevalence setting of Papua New Guinea for over three decades. Microbial Genomics.

[42] Smith C, Halse TA, Shea J, Modestil H, Fowler RC (2020). Assessing nanopore sequencing for clinical diagnostics: a comparison of Next-Generation Sequencing (NGS) methods for *Mycobacterium tuberculosis*. J Clin Microbiol.

[43] Peker N, Schuele L, Kok N, Terrazos M, Neuenschwander SM (2021). Evaluation of whole-genome sequence data analysis approaches for short- and long-read sequencing of *Mycobacterium tuberculosis*. Microbial Genomics.

[44] Merker M, Rasigade J-P, Barbier M, Cox H, Feuerriegel S (2022). Transcontinental spread and evolution of *Mycobacterium tuberculosis* W148 European/Russian clade toward extensively drug resistant tuberculosis. Nat Commun.

[45] Finci I, Albertini A, Merker M, Andres S, Bablishvili N (2022). Investigating resistance in clinical *Mycobacterium tuberculosis* complex isolates with genomic and phenotypic antimicrobial susceptibility testing: a multicentre observational study. Lancet Microbe.

[46] Roberts LW, Malone KM, Hunt M, Joseph L, Wintringer P (2022). Repeated evolution of bedaquiline resistance in *Mycobacterium tuberculosis* is driven by truncation of mmpR5. Microbiology.

[47] Di Marco F, Spitaleri A, Battaglia S, Batignani V, Cabibbe AM (2023). Advantages of long- and short-reads sequencing for the hybrid investigation of the *Mycobacterium tuberculosis* genome. Front Microbiol.

[48] Lempens P, Decroo T, Aung KJM, Hossain MA, Rigouts L (2020). Initial resistance to companion drugs should not be considered an exclusion criterion for the shorter multidrug-resistant tuberculosis treatment regimen. Int J Infect Dis.

[49] Lempens P, Meehan CJ, Vandelannoote K, Fissette K, de Rijk P (2018). Isoniazid resistance levels of *Mycobacterium tuberculosis* can largely be predicted by high-confidence resistance-conferring mutations. Sci Rep.

[50] Steinig E, Coin L (2022). Nanoq: ultra-fast quality control for nanopore reads. J Open Source Softw.

[51] Chen S, Zhou Y, Chen Y, Gu J (2018). fastp: an ultra-fast all-in-one FASTQ preprocessor. Bioinformatics.

[52] Hunt M, Letcher B, Malone KM, Nguyen G, Hall MB (2022). Minos: variant adjudication and joint genotyping of cohorts of bacterial genomes. Genome Biol.

[53] Virtanen P, Gommers R, Oliphant TE, Haberland M, Reddy T (2020). Author Correction: SciPy 1.0: fundamental algorithms for scientific computing in Python. Nat Methods.

[54] Chen Y, Takiff HE, Gao Q (2023). Phenotypic instability of *Mycobacterium tuberculosis* strains harbouring clinically prevalent drug-resistant mutations. Lancet Microbe.

[55] Sirgel FA, Warren RM, Streicher EM, Victor TC, van Helden PD (2012). embB306 mutations as molecular indicators to predict ethambutol susceptibility in *Mycobacterium tuberculosis*. Chemotherapy.

[56] Huo F, Ma Y, Li S, Xue Y, Shang Y (2020). Specific gyrA gene mutations correlate with high prevalence of discordant levofloxacin resistance in *Mycobacterium tuberculosis* isolates from Beijing, China. J Mol Diagn.

[57] Brankin AE, Fowler PW (2023). Inclusion of minor alleles improves catalogue-based prediction of fluoroquinolone resistance in *Mycobacterium tuberculosis*. JAC Antimicrob Resist.

[58] Nimmo C, Brien K, Millard J, Grant AD, Padayatchi N (2020). Dynamics of within-host *Mycobacterium tuberculosis* diversity and heteroresistance during treatment. EBioMedicine.

[59] Perdigão J, Gomes P, Miranda A, Maltez F, Machado D (2020). Using genomics to understand the origin and dispersion of multidrug and extensively drug resistant tuberculosis in Portugal. Sci Rep.

[60] Singh A, Singh A, Grover S, Pandey B, Kumari A (2018). Wild-type catalase peroxidase vs G279D mutant type: Molecular basis of Isoniazid drug resistance in *Mycobacterium tuberculosis*. Gene.

[61] Vaziri F, Kohl TA, Ghajavand H, Kargarpour Kamakoli M, Merker M (2019). Genetic diversity of multi- and extensively drug-resistant *Mycobacterium tuberculosis* isolates in the Capital of Iran, revealed by whole-genome sequencing. J Clin Microbiol.

[62] de Lourdes do Carmo Guimarães Diniz J, von Groll A, Unis G, Dalla-Costa ER, Rosa Rossetti ML (2021). Whole-genome sequencing as a tool for studying the microevolution of drug-resistant serial *Mycobacterium tuberculosis* isolates. Tuberculosis.

[63] Altamirano M, Marostenmaki J, Wong A, FitzGerald M, Black WA (1994). Mutations in the catalase-peroxidase gene from isoniazid-resistant *Mycobacterium tuberculosis* isolates. J Infect Dis.

[64] Ferrazoli L, Palaci M, Telles MA, Ueki SY, Kritski A (1995). Catalase expression, katG, and MIC of isoniazid for *Mycobacterium tuberculosis* isolates from SãO Paulo, Brazil. J Infect Dis.

[65] Ramaswamy SV, Reich R, Dou S-J, Jasperse L, Pan X (2003). Single nucleotide polymorphisms in genes associated with isoniazid resistance in *Mycobacterium tuberculosis*. Antimicrob Agents Chemother.

[66] Zhang Y, Heym B, Allen B, Young D, Cole S (1992). The catalase—peroxidase gene and isoniazid resistance of *Mycobacterium tuberculosis*. Nature.

[67] Martinez E, Holmes N, Jelfs P, Sintchenko V (2015). Genome sequencing reveals novel deletions associated with secondary resistance to pyrazinamide in MDR *Mycobacterium tuberculosis*. J Antimicrob Chemother.

[68] Garrison E, Sirén J, Novak AM, Hickey G, Eizenga JM (2018). Variation graph toolkit improves read mapping by representing genetic variation in the reference. Nat Biotechnol.

[69] Liao W-W, Asri M, Ebler J, Doerr D, Haukness M (2023). A draft human pangenome reference. Nature.

[70] Samarakoon H, Punchihewa S, Senanayake A, Hammond JM, Stevanovski I (2020). Genopo: a nanopore sequencing analysis toolkit for portable Android devices. Commun Biol.

[71] Gómez-González PJ, Perdigao J, Gomes P, Puyen ZM, Santos-Lazaro D (2021). Genetic diversity of candidate loci linked to *Mycobacterium tuberculosis* resistance to bedaquiline, delamanid and pretomanid. Sci Rep.

[72] Schena E, Nedialkova L, Borroni E, Battaglia S, Cabibbe AM (2016). Delamanid susceptibility testing of *Mycobacterium tuberculosis* using the resazurin microtitre assay and the BACTEC^TM^ MGIT^TM^ 960 system. J Antimicrob Chemother.

[73] Gomes LC, Campino S, Marinho CRF, Clark TG, Phelan JE (2021). Whole genome sequencing reveals large deletions and other loss of function mutations in *Mycobacterium tuberculosis* drug resistance genes. Microb Genom.

[74] De Maio F, Cingolani A, Bianco DM, Salustri A, Palucci I (2021). First description of the katG gene deletion in a *Mycobacterium tuberculosis* clinical isolate and its impact on the mycobacterial fitness. Int J Med Microbiol.

[75] Ang MLT, Zainul Rahim SZ, de Sessions PF, Lin W, Koh V (2017). EthA/R-independent killing of *Mycobacterium tuberculosis* by ethionamide. Front Microbiol.

[76] Allix-Béguec C, Arandjelovic I, Bi L, Beckert P, Bonnet M (2018). Prediction of susceptibility to first-line tuberculosis drugs by DNA sequencing. N Engl J Med.

[77] Köser CU, Cirillo DM, Miotto P (2020). How to optimally combine genotypic and phenotypic drug susceptibility testing methods for pyrazinamide. Antimicrob Agents Chemother.

[78] Yadon AN, Maharaj K, Adamson JH, Lai Y-P, Sacchettini JC (2017). A comprehensive characterization of PncA polymorphisms that confer resistance to pyrazinamide. Nat Commun.

[79] Park S-C, Lee K, Kim YO, Won S, Chun J (2019). Large-scale genomics reveals the genetic characteristics of seven species and importance of phylogenetic distance for estimating pan-genome size. Front Microbiol.

[80] Zwyer M, Çavusoglu C, Ghielmetti G, Pacciarini ML, Scaltriti E (2021). A new nomenclature for the livestock-associated *Mycobacterium tuberculosis* complex based on phylogenomics. Open Res Europe.

