# Supplementary material for: Drug resistance prediction for Mycobacterium tuberculosis with reference graphs
Source: Microb Genom. 2023 Aug 8;9(8):mgen001081. doi: 10.1099/mgen.0.001081 (PMC10483414; doi:10.1099/mgen.0.001081)
Supplement: Supplementary material 1 [file mgen-9-1081-s001.pdf]

Supplementary material

Contents

S1 Too many mutations in the reference graph degrades performance **2**

S2 Choice of graph density and variant filters **2**

S3 Example DrPRG prediction output **6**

S4 Sensitivity and specificity performance **8**

References **11**

List of Tables

S1 Comparison of DrPRG pyrazinamide sensitivity and specificity on Illumina data when using a selection of *pncA* mutations versus using all grade 1 and 2 *pncA* mutations in the WHO catalogue. FN=number of false negatives, R=number of resistant isolates, FP=number of false positives, S=number of susceptible isolates, CI=confidence interval. 2

S2 Comparison of resistance predictions from Illumina data for three tools. FN=number of false negatives, R=number of resistant isolates, FP=number of false positives, S=number of susceptible isolates, CI=confidence interval. 9

S3 Comparison of resistance predictions from Nanopore data for three programs. FN=number of false negatives, R=number of resistant isolates, FP=number of false positives, S=number of susceptible isolates, CI=confidence interval. 10

S1 Too many mutations in the reference graph degrades performance

As has been noted elsewhere ([1, 2], and Section S2), adding mutations to build a reference graph reaches a saturation point, beyond which both computational and accuracy performance degrade. As such, we chose not to build our reference graph from all known resistance-conferring mutations. To make this point explicit and to quantify just how poorly DrPRG's performance is when using all mutations, we rebuilt the *M. tuberculosis* reference graph including all WHO catalogue[3] grade 1 and 2 non-frameshift mutations in *pncA* - a total of 176 mutations, 19 of which were already in the graph.

After rerunning DrPRG on all Illumina samples, the results are quite dramatic, as shown in Table S1. When incorporating all non-frameshift *pncA* mutations into the graph, 18049/18050 pyrazinamide-susceptible samples are called resistant, leaving a single true negative call and a specificity of 0.0% (compared to 97.2% when using the reference graph in Table S2). In addition, adding all of these non-frameshift *pncA* mutations leads to a missed resistant call (false negative) in only six of the 3836 pyrazinamide-resistant sample giving a sensitivity of 99.8% (compared to 79.8% when using the reference graph in Table S2).

Table S1: Comparison of DrPRG pyrazinamide sensitivity and specificity on Illumina data when using a selection of *pncA* mutations versus using all grade 1 and 2 *pncA* mutations in the WHO catalogue. FN=number of false negatives, R=number of resistant isolates, FP=number of false positives, S=number of susceptible isolates, CI=confidence interval.

pncA mutations	FN(R)	FP(S)	Sensitivity (95% CI)	Specificity (95% CI)
19	776(3836)	497(18050)	79.8% (78.5-81.0%)	97.2% (97.0-97.5%)
176	6(3836)	18049(18050)	99.8% (99.7-99.9%)	0.0% (0.0-0.0%)

S2 Choice of graph density and variant filters

S2.1 Construction of *M. tuberculosis* reference graphs

The text and figure in this section are taken from [2] under the terms of the CC BY 4.0 license. The text is adapted to fit the context of this manuscript, but with no change in meaning or interpretation.

Pandora requires a pan-genome reference graph (PanRG) in order to operate; we chose to construct a reference PanRG based on the *M. tuberculosis* reference genome, H37Rv (accession NC_000962.3). We add variants sampled from 15,211 global *M. tuberculosis* isolates gathered by the CRyPTIC consortium [4]. We sampled at two different rates to evaluate how varying complexity of PanRGs affect variant-calling precision and recall.

To ensure the reference PanRG is not biased towards a particular lineage, we first split the global CRyPTIC VCF into separate lineage VCFs. In addition, we

include variant calls from 14 high-quality *M. tuberculosis* assemblies, representing lineages 1-7 [5, 6]. The two PanRG complexities we construct were termed "sparse" and "dense". From each lineage VCF, we took a random subsample of 20 and 200 samples and combined them into single sparse and dense VCFs, respectively. Note, we use the same fixed random seed for the subsampling to ensure the sparse PanRG is a subset of the dense PanRG. Finally, we filtered the resulting VCFs to remove any positions with no alternate allele calls or that failed the filtering applied by the CRyPTIC pipeline `clockwork`. One exception is that we did not remove positions that overlap a genome mask of repetitive regions in H37Rv [7].

The reference PanRG that Pandora uses is actually a collection of local PRGs (loci). These loci are effectively partitions of the original genome; one can partition based on any criteria. We chose to split the H37Rv genome based on the genomic features outlined in the accompanying General Feature Format (GFF) file from the NCBI database. We also retain the segments *between* the features - so-called intergenic regions (IGRs). We combine genomic features with overlapping coordinates (e.g., transcribed on opposite strands or different reading frames) into a single locus and join any locus (feature or IGR) shorter than 500bp with its 3' neighbour. By building the reference PanRG in this manner, we ensure representation of every position in the H37Rv genome amongst all loci. We then remove any locus with 30% or more of its positions overlapping the H37Rv genome mask mentioned above [7].

We form the sparse and dense PanRGs by applying the variants from the respective VCF to the template (reference) sequence of each locus; for each position in the VCF, we infer the locus it corresponds to. We then take all (called) alternate alleles and create a sequence for each; that is, the template sequence, with the reference allele replaced by the alternate allele. Note, we disregard any indels longer than 20bp or that span a locus boundary. Finally, all of these sequences are pooled into a single fasta file for each locus.

The multi-sequence fasta file for each locus is then subjected to multiple sequence alignment (MSA) using MAFFT (v7.471; [8]). We use the accurate global alignment setting, G-INS-i [9], with default parameters, using the `ginsi` script provided with MAFFT. The resulting MSA is then converted to a Pandora-compatible PRG using the `make_prg` program (v0.1.1; [10]) with a maximum nesting level of 5 and minimum match length of 7. All of the local PRGs are then combined into a single PanRG file and indexed with Pandora[10] using a k-mer size of 15 and window size of 14. In the end, we have two PanRG files - sparse and dense.

S2.2 Validating variant calls

We evaluate the precision and recall of SNPs using `varifier` with a flank length of 100bp [11]. The samples we evaluated are those seven with PacBio assemblies that passed QC from [12] - these also have matched Illumina and Nanopore data. As a truth genome for each, we use the unpolished PacBio assembly, along with a mask for low-quality regions. These low-quality regions were identified

by aligning the sample's Illumina reads to the assembly with `bwa mem`[13] and flagging any position with less than 10 reads mapping to it or less than 90% agreement.

Single-reference variant calling

Conventional single-reference Illumina variant calls were performed with the COMPASS pipeline (https://github.com/oxfordmmm/CompassCompact) used by Public Health England. Briefly, reads are mapped to H37Rv, and `samtools mpileup` is used to identify SNPs [14]. SNPs are filtered based on the following criteria: i) must have at least five high-quality supporting reads, ii) must have at least one read in each direction, iii) 75% of reads must be high-quality, iv) the diploid genotype must be homozygous, v) fraction of reads supporting the major allele must be at least 90%. In addition, any SNPs falling within masked sites - as defined by aligning the H37Rv to itself and identifying repetitive regions [7] - are excluded.

Reference graph variant calling

When assessing the best filters for increasing the precision of variant calls from Pandora, we are also interested in determining whether PanRG density has a noticeable impact on performance. Therefore, we use the sparse and dense PanRGs from Section S2.1 and look at the precision and recall these produce for the same filters.

For each sample, we discover novel variants using the `discover` command of Pandora (version 0.8.0). We use default parameters, except limiting the number of novel variants from a candidate region to 10. Novel variants are added to the original MSAs from Section S2.1 with the `--add` routine in MAFFT [15]. Next, `make_prg` is run on the subsequent alignments, and the resulting updated PanRG is indexed with Pandora. Finally, the `map` routine of Pandora genotypes a sample's reads and produces a VCF. To be able to compare the Pandora VCF to the truth assemblies, we instruct Pandora to output coordinates with respect to the H37Rv reference sequence for each locus. Running Pandora in this way leads to some alleles being quite long and redundant, so we use `bcftools norm` to trim unused alleles and reduce variants down to their most succinct representation[16].

We apply four filters to the Pandora variant calls. First, there must be at least 3 reads supporting the called allele. Second, we keep positions with a strand bias of at least 1%, which is the lowest depth on the forward or reverse strand divided by the total depth. This is a somewhat different definition of strand bias to that used in human genetics [17]; our definition is testing whether there are significantly more reads on one strand. For example, in this definition, if the forward and reverse strand have read depths of 1 and 9, respectively, the strand bias is 10%. Therefore, this example position would not be filtered out. Third, a genotype confidence score no less than 5. This score is the difference between the log-likelihoods of the called allele and the next most likely allele.

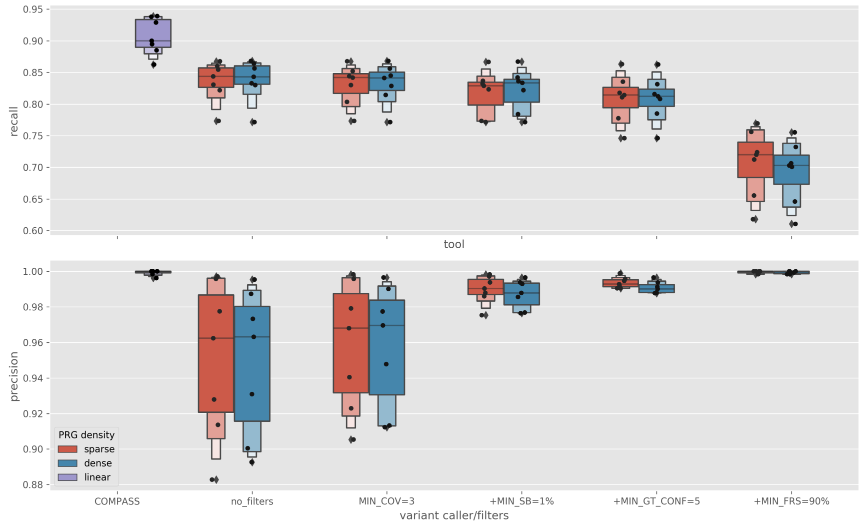

Figure S1: Precision (bottom) and recall (top) of SNPs for COMPASS (purple) and Pandora with sparse (red) and dense (blue) PanRGs. The Pandora boxes start with no filters on the left, with each box moving to the right adding a filter to the previous box. The COMPASS box is a reference to the precision and recall of Illumina variant calls. Linear PRG density refers to the fact that COMPASS uses a single, linear reference genome as opposed to Pandora, which uses a genome graph. The black points refer to single data points for the seven samples used. MIN_COV=minimum depth of coverage;MIN_SB=minimum strand bias;MIN_GT_CONF=minimum genotype confidence score;MIN_FRS=minimum fraction of read support.

Source: [2] under the terms of the CC BY 4.0 license

Fourth, the fraction of reads supporting the called allele (FRS) must be at least 90%.

The results of incrementally applying these filters, along with no filters and COMPASS, are shown in Figure S1. Of the two PanRGs used, Pandora's best median precision (100%) is with the sparse PanRG and all filters applied. With all filters, the sparse PanRG leads to a median recall of 71.99%. When compared to the COMPASS median precision and recall values of 100% and 92.58%, respectively, Pandora produces Nanopore SNP calls with equivalent precision to Illumina, but with 20.59% less recall. Part of the recall disparity between Pandora and COMPASS is explained by the masking of loci in the reference graph (Section S2.1).

In nearly every filtering combination, the sparse PanRG lead to higher recall *and* precision, albeit marginally. As a result, when building the drug resistance reference graph for DrPRG we opted for a sparse graph with only 20 samples from each lineage (see Constructing a resistance-specific reference graph and index in the main manuscript). Additionally, we chose only the first two filters

(minimum depth 3 and minimum strand bias 1%) when using Pandora in DrPRG as we found in testing that the FRS and genotype confidence filters were causing DrPRG to miss a large number of minor allele calls.

S3 Example DrPRG prediction output

This is a trimmed (toy) example of the JSON output from DrPRG.


```
{
  "genes": {
    "absent": [
      "ahpC"
    ],
    "present": [
      "embA",
      "embB",
      "ethA",
      "fabG1",
      "gid",
      "gyrA",
      "gyrB",
      "inhA",
      "katG",
      "pncA",
      "rpoB",
      "rrs"
    ]
  },
  "sample": "toy",
  "susceptibility": {
    "Amikacin": {
      "evidence": [
        {
          "gene": "rrs",
          "residue": "DNA",
          "variant": "A1401X",
          "vcfid": "b815ed3f"
        }
      ],
      "predict": "F"
    },
    "Ethambutol": {
      "evidence": [
        {
          "gene": "embB",
```

```
          "residue": "PROT",
          "variant": "M306I",
          "vcfid": "a290b118"
        }
      ],
      "predict": "r"
    },
    "Ethionamide": {
      "evidence": [
        {
          "gene": "ethA",
          "residue": "PROT",
          "variant": "A381P",
          "vcfid": "169f75d4"
        }
      ],
      "predict": "U"
    },
    "Isoniazid": {
      "evidence": [
        {
          "gene": "fabG1",
          "residue": "DNA",
          "variant": "G-17T",
          "vcfid": "de9b689e"
        },
        {
          "gene": "katG",
          "residue": "PROT",
          "variant": "S315T",
          "vcfid": "acaa8ca2"
        }
      ],
      "predict": "R"
    },
    "Levofloxacin": {
      "evidence": [],
      "predict": "S"
    }
  },
  "version": {
    "drprg": "0.1.1",
    "index": "20230308"
  }
}
```

The keys of the JSON are:

- `genes`: This contains a list of genes in the index reference graph which are present and absent.
- `sample`: The value passed to the `-s/--sample` option.
- `susceptibility`: The keys of this entry are the drugs in the index catalogue. Each drug's entry contains `evidence` (Section S3.2) supporting the value in the `predict` (Section S3.1) section.
- `version`: the version of DrPRG and the index used.

S3.1 Predict

The `predict` entry for a drug is the resistance prediction for the sample. Possible values are:

- `S`: susceptible. This is the default prediction. If no mutations are detected for the sample, it is assumed to be susceptible.
- `F`: failed. Genotyping failed for one or more mutations for this drug. See the prediction VCF for more information.
- `U`: unknown. One or more mutations that are not present in the index catalogue were detected in a gene associated with this drug.
- `R`: resistant. One or more mutations from the index catalogue that confer resistance were detected.
- `u` or `r`: The same as the uppercase versions, but the mutation(s) were detected in a minor allele.

S3.2 Evidence

This is a list of the mutations supporting the prediction. The residue is one of `DNA` or `PROT` indicating whether the mutation describes a nucleotide or amino acid change, respectively. The variant is of the form `<ref><pos><alt>`; where `<ref>` is the reference sequence at position `<pos>` and `<alt>` is the nucleotide/amino acid the reference is changed to. See the catalogue documentation for more information. The `vcfid` is the value in the prediction VCF ID column for this mutation, making it easier to find a mutation in the VCF.

S4 Sensitivity and specificity performance

Table S2: Comparison of resistance predictions from Illumina data for three tools. FN=number of false negatives, R=number of resistant isolates, FP=number of false positives, S=number of susceptible isolates, CI=confidence interval.

Drug	Tool	FN(R)	FP(S)	Sensitivity (95% CI)	Specificity (95% CI)
Isoniazid	DrPRG	1005(14671)	586(25769)	93.1% (92.7-93.5%)	97.7% (97.5-97.9%)
	Mykrobe	1076(14671)	561(25769)	92.7% (92.2-93.1%)	**97.8% (97.6-98.0%)**
	TBProfiler	989(14671)	649(25769)	**93.3% (92.8-93.7%)**	97.5% (97.3-97.7%)
Rifampicin	DrPRG	426(11776)	620(28292)	96.4% (96.0-96.7%)	97.8% (97.6-98.0%)
	Mykrobe	523(11776)	604(28292)	95.6% (95.2-95.9%)	**97.9% (97.7-98.0%)**
	TBProfiler	370(11776)	788(28292)	**96.9% (96.5-97.2%)**	97.2% (97.0-97.4%)
Ethambutol	DrPRG	595(6014)	2290(27011)	90.1% (89.3-90.8%)	91.5% (91.2-91.8%)
	Mykrobe	631(6014)	2265(27011)	89.5% (88.7-90.3%)	**91.6% (91.3-91.9%)**
	TBProfiler	578(6014)	2293(27011)	**90.4% (89.6-91.1%)**	91.5% (91.2-91.8%)
Pyrazinamide	DrPRG	776(3836)	497(18050)	79.8% (78.5-81.0%)	97.2% (97.0-97.5%)
	Mykrobe	798(3836)	449(18050)	79.2% (77.9-80.5%)	**97.5% (97.3-97.7%)**
	TBProfiler	680(3836)	513(18050)	**82.3% (81.0-83.4%)**	97.2% (96.9-97.4%)
Levofloxacin	DrPRG	268(3109)	355(14867)	**91.4% (90.3-92.3%)**	97.6% (97.4-97.8%)
	Mykrobe	299(3109)	330(14867)	90.4% (89.3-91.4%)	**97.8% (97.5-98.0%)**
	TBProfiler	276(3109)	356(14867)	91.1% (90.1-92.1%)	97.6% (97.3-97.8%)
Moxifloxacin	DrPRG	178(2260)	1135(14698)	**92.1% (90.9-93.2%)**	92.3% (91.8-92.7%)
	Mykrobe	207(2260)	1113(14698)	90.8% (89.6-92.0%)	**92.4% (92.0-92.8%)**
	TBProfiler	182(2260)	1141(14698)	91.9% (90.8-93.0%)	92.2% (91.8-92.7%)
Ofloxacin	DrPRG	138(781)	69(6008)	**82.3% (79.5-84.8%)**	98.9% (98.5-99.1%)
	Mykrobe	147(781)	62(6008)	81.2% (78.3-83.8%)	**99.0% (98.7-99.2%)**
	TBProfiler	138(781)	65(6008)	**82.3% (79.5-84.8%)**	98.9% (98.6-99.2%)
Amikacin	DrPRG	269(1866)	224(18737)	**85.6% (83.9-87.1%)**	98.8% (98.6-99.0%)
	Mykrobe	359(1866)	192(18737)	80.8% (78.9-82.5%)	**99.0% (98.8-99.1%)**
	TBProfiler	270(1866)	227(18737)	85.5% (83.9-87.1%)	98.8% (98.6-98.9%)
Capreomycin	DrPRG	292(1300)	299(13039)	**77.5% (75.2-79.7%)**	97.7% (97.4-98.0%)
	Mykrobe	367(1300)	263(13039)	71.8% (69.3-74.1%)	**98.0% (97.7-98.2%)**
	TBProfiler	293(1300)	305(13039)	77.5% (75.1-79.7%)	97.7% (97.4-97.9%)
Kanamycin	DrPRG	361(2213)	319(18382)	83.7% (82.1-85.2%)	98.3% (98.1-98.4%)
	Mykrobe	445(2213)	298(18382)	79.9% (78.2-81.5%)	**98.4% (98.2-98.6%)**
	TBProfiler	357(2213)	322(18382)	**83.9% (82.3-85.3%)**	98.2% (98.0-98.4%)
Streptomycin	DrPRG	787(5365)	681(10179)	85.3% (84.4-86.3%)	93.3% (92.8-93.8%)
	Mykrobe	906(5365)	676(10179)	83.1% (82.1-84.1%)	93.4% (92.9-93.8%)
	TBProfiler	780(5365)	661(10179)	**85.5% (84.5-86.4%)**	**93.5% (93.0-94.0%)**
Ethionamide	DrPRG	733(2961)	1023(11360)	**75.2% (73.7-76.8%)**	91.0% (90.5-91.5%)
	Mykrobe	847(2961)	985(11360)	71.4% (69.7-73.0%)	**91.3% (90.8-91.8%)**
	TBProfiler	844(2961)	995(11360)	71.5% (69.8-73.1%)	91.2% (90.7-91.7%)
Linezolid	DrPRG	104(152)	30(10915)	**31.6% (24.7-39.3%)**	99.7% (99.6-99.8%)
	Mykrobe	105(152)	29(10915)	30.9% (24.1-38.7%)	**99.7% (99.6-99.8%)**
	TBProfiler	104(152)	31(10915)	**31.6% (24.7-39.3%)**	99.7% (99.6-99.8%)
Delamanid	DrPRG	111(116)	3(8154)	**4.3% (1.9-9.7%)**	100.0% (99.9-100.0%)
	Mykrobe	111(116)	2(8154)	**4.3% (1.9-9.7%)**	**100.0% (99.9-100.0%)**
	TBProfiler	111(116)	2(8154)	**4.3% (1.9-9.7%)**	**100.0% (99.9-100.0%)**

Table S3: Comparison of resistance predictions from Nanopore data for three programs. FN=number of false negatives, R=number of resistant isolates, FP=number of false positives, S=number of susceptible isolates, CI=confidence interval.

Drug	Tool	FN(R)	FP(S)	Sensitivity (95% CI)	Specificity (95% CI)
Isoniazid	DrPRG	9(60)	5(48)	85.0% (73.9-91.9%)	89.6% (77.8-95.5%)
	Mykrobe	9(60)	4(48)	85.0% (73.9-91.9%)	91.7% (80.4-96.7%)
	TBProfiler	9(60)	3(48)	85.0% (73.9-91.9%)	**93.8% (83.2-97.9%)**
Rifampicin	DrPRG	5(57)	1(44)	91.2% (81.1-96.2%)	97.7% (88.2-99.6%)
	Mykrobe	5(57)	1(44)	91.2% (81.1-96.2%)	97.7% (88.2-99.6%)
	TBProfiler	5(57)	1(44)	91.2% (81.1-96.2%)	97.7% (88.2-99.6%)
Ethambutol	DrPRG	4(21)	15(77)	**81.0% (60.0-92.3%)**	80.5% (70.3-87.8%)
	Mykrobe	4(21)	15(77)	**81.0% (60.0-92.3%)**	80.5% (70.3-87.8%)
	TBProfiler	5(21)	15(77)	76.2% (54.9-89.4%)	80.5% (70.3-87.8%)
Ofloxacin	DrPRG	0(11)	4(79)	100.0% (74.1-100.0%)	94.9% (87.7-98.0%)
	Mykrobe	0(11)	4(79)	100.0% (74.1-100.0%)	94.9% (87.7-98.0%)
	TBProfiler	0(11)	3(79)	100.0% (74.1-100.0%)	**96.2% (89.4-98.7%)**
Amikacin	DrPRG	0(14)	3(81)	100.0% (78.5-100.0%)	96.3% (89.7-98.7%)
	Mykrobe	0(14)	3(81)	100.0% (78.5-100.0%)	96.3% (89.7-98.7%)
	TBProfiler	0(14)	3(81)	100.0% (78.5-100.0%)	96.3% (89.7-98.7%)
Capreomycin	DrPRG	1(4)	1(54)	75.0% (30.1-95.4%)	98.1% (90.2-99.7%)
	Mykrobe	1(4)	1(54)	75.0% (30.1-95.4%)	98.1% (90.2-99.7%)
	TBProfiler	1(4)	1(54)	75.0% (30.1-95.4%)	98.1% (90.2-99.7%)
Kanamycin	DrPRG	0(3)	1(55)	100.0% (43.9-100.0%)	98.2% (90.4-99.7%)
	Mykrobe	0(3)	1(55)	100.0% (43.9-100.0%)	98.2% (90.4-99.7%)
	TBProfiler	0(3)	1(55)	100.0% (43.9-100.0%)	98.2% (90.4-99.7%)
Streptomycin	DrPRG	3(10)	14(83)	70.0% (39.7-89.2%)	83.1% (73.7-89.7%)
	Mykrobe	3(10)	27(83)	70.0% (39.7-89.2%)	67.5% (56.8-76.6%)
	TBProfiler	3(10)	12(83)	70.0% (39.7-89.2%)	**85.5% (76.4-91.5%)**
Ethionamide	DrPRG	0(5)	1(9)	100.0% (56.6-100.0%)	88.9% (56.5-98.0%)
	Mykrobe	0(5)	1(9)	100.0% (56.6-100.0%)	88.9% (56.5-98.0%)
	TBProfiler	0(5)	1(9)	100.0% (56.6-100.0%)	88.9% (56.5-98.0%)

References

[1] Jacob Pritt, Nae-Chyun Chen, and Ben Langmead. "FORGe: prioritizing variants for graph genomes". In: *Genome Biology* 19.1 (2018), p. 220. DOI: 10.1186/s13059-018-1595-x.

[2] Michael B. Hall. "Examining bacterial variation with genome graphs and Nanopore sequencing". PhD thesis. University of Cambridge, 2022. DOI: 10.17863/CAM.81350.

[3] Timothy M Walker et al. "The 2021 WHO catalogue of Mycobacterium tuberculosis complex mutations associated with drug resistance: a genotypic analysis". In: *The Lancet Microbe* (2022). ISSN: 2666-5247. DOI: 10.1016/s2666-5247(21)00301-3.

[4] Alice Brankin et al. "A data compendium of Mycobacterium tuberculosis antibiotic resistance". In: *bioRxiv* (2021). DOI: 10.1101/2021.09.14.460274.

[5] Álvaro Chiner-Oms et al. "Genome-wide mutational biases fuel transcriptional diversity in the Mycobacterium tuberculosis complex". In: *Nature Communications* 10.1 (2019), p. 3994. DOI: 10.1038/s41467-019-11948-6.

[6] Brice Letcher, Martin Hunt, and Zamin Iqbal. "Gramtools enables multiscale variation analysis with genome graphs". In: *Genome Biology* 22.1 (2021), p. 259. DOI: 10.1186/s13059-021-02474-0.

[7] Timothy M Walker et al. "Assessment of Mycobacterium tuberculosis transmission in Oxfordshire, UK, 2007–12, with whole pathogen genome sequences: an observational study". In: *The Lancet Respiratory Medicine* 2.4 (2014), pp. 285–292. ISSN: 2213-2600. DOI: 10.1016/s2213-2600(14)70027-x.

[8] Tsukasa Nakamura et al. "Parallelization of MAFFT for large-scale multiple sequence alignments". In: *Bioinformatics* 34.14 (2018), pp. 2490–2492. ISSN: 1367-4803. DOI: 10.1093/bioinformatics/bty121.

[9] Kazutaka Katoh and Daron M. Standley. "A simple method to control over-alignment in the MAFFT multiple sequence alignment program". In: *Bioinformatics* 32.13 (2016), pp. 1933–1942. ISSN: 1367-4803. DOI: 10.1093/bioinformatics/btw108.

[10] Rachel M. Colquhoun et al. "Pandora: nucleotide-resolution bacterial pan-genomics with reference graphs". In: *Genome Biology* 22.1 (Sept. 2021), p. 267. ISSN: 1474-760X. DOI: 10.1186/s13059-021-02473-1.

[11] Martin Hunt et al. "Minos: variant adjudication and joint genotyping of cohorts of bacterial genomes". In: *Genome Biology* 23.1 (July 2022), p. 147. ISSN: 1474-760X. DOI: 10.1186/s13059-022-02714-x. (Visited on 04/22/2023).

[12] Michael B. Hall et al. "Evaluation of Nanopore sequencing for Mycobacterium tuberculosis drug susceptibility testing and outbreak investigation: a genomic analysis". English. In: *The Lancet Microbe* 0.0 (Dec. 2022). Publisher: Elsevier. ISSN: 2666-5247. DOI: 10.1016/S2666-5247(22)00301-9. (Visited on 01/05/2023).

[13] Heng Li. *Aligning sequence reads, clone sequences and assembly contigs with BWA-MEM*. 2013. arXiv: 1303.3997 [q-bio.GN].

[14] Heng Li et al. "The Sequence Alignment/Map format and SAMtools". In: *Bioinformatics* 25.16 (2009), pp. 2078–2079. ISSN: 1367-4803. DOI: 10.1093/bioinformatics/btp352.

[15] Kazutaka Katoh and Martin C. Frith. "Adding unaligned sequences into an existing alignment using MAFFT and LAST". In: *Bioinformatics* 28.23 (2012), pp. 3144–3146. ISSN: 1367-4803. DOI: 10.1093/bioinformatics/bts578.

[16] Petr Danecek et al. "Twelve years of SAMtools and BCFtools". In: *GigaScience* 10.2 (2021), giab008. ISSN: 2047-217X. DOI: 10.1093/gigascience/giab008.

[17] Yan Guo et al. "The effect of strand bias in Illumina short-read sequencing data". In: *BMC genomics* 13.1 (2012), pp. 1–11. DOI: 10.1186/1471-2164-13-666.
